# Feasibility of indocyanine green fluorescence imaging for intraoperative identification of parathyroid glands during thyroid surgery

**DOI:** 10.1002/hed.25451

**Published:** 2018-12-11

**Authors:** Jacqueline van den Bos, Lottie van Kooten, Sanne M. E. Engelen, Tim Lubbers, Laurents P. S. Stassen, Nicole D. Bouvy

**Affiliations:** ^1^ Department of Surgery Maastricht University Medical Center Maastricht The Netherlands; ^2^ Faculty of Health Medicine and Life Sciences Maastricht University Maastricht The Netherlands

**Keywords:** indocyanine green, near‐infrared fluorescence imaging, NIRF, parathyroid gland, thyroidectomy

## Abstract

**Background:**

This study assessed the feasibility of near‐infrared fluorescence imaging with indocyanine green (ICG) to identify the parathyroid glands (PGs) intraoperatively and to assess their perfusion after thyroid resection.

**Methods:**

Patients undergoing elective thyroidectomy were enrolled in this prospective study. An intravenous bolus of 7.5 mg ICG was administered twice: the first bolus to identify the PGs before resection of the thyroid and the second to assess vascularization of the PGs after resection.

**Results:**

A total of 30 operations in 26 patients were included. In 17 surgeries (56.7%), fluorescence imaging was of added value, especially to confirm the presence of a suspected PG. No intraoperative or postoperative complications occurred because of the use of ICG.

**Conclusion:**

Near‐infrared fluorescence imaging with the use of ICG for intraoperative identification of the PGs and the assessment of its vascularization is feasible and safe and can provide more certainty about the location of the PGs.

## 1 INTRODUCTION

Iatrogenic injury of the parathyroid glands (PGs) is the most common complication after total thyroidectomy.^1^ Damage of the PGs can cause hypoparathyroidism, which results in hypocalcaemia. The reported incidence of hypocalcaemia after total thyroidectomy varies widely: between 1.6% and 50%.^2^, ^3^, ^4^ Hypocalcaemia can result in increased morbidity, including cardiac arrhythmias and tetany leading to prolonged hospitalization and even death.^5^ To avoid parathyroid injury, it is essential to identify the PGs and preserve their blood supply. However, this can be challenging due to their small size and the fact that they can be difficult to distinguish from their surrounding tissues. Intraoperative guidance to help identify and assess the PGs during thyroid surgery may prevent surgical damage and thus provide a better postoperative outcome and quality of life.

A few intraoperative imaging techniques have been described. Dudley^6^ was the first to use intravenous administration of methylene blue (MB) to stain parathyroid tissue in vivo in 1971. After publication of this study, MB has been implemented by other surgeons.^7^ The chance of complications due to intravenous administration of MB, including neurotoxicity and acute MB‐induced phototoxicity, pain at infusion site and nausea is a major drawback of this technique.^7^, ^8^, ^9^, ^10^ To avoid these complications due to a high dose of MB, a lower dose in combination with near‐infrared fluorescence (NIRF) imaging was investigated.^11^ However, in analogy with the use of MB for staining without NIRF imaging, this technique mainly visualizes abnormal PGs. To avoid iatrogenic damage, visualization of normal PGs is essential.

A technique in which both abnormal and normal PGs can be identified is fluorescence imaging with 5‐aminolevulinic acid.^12^ However, this technique also has its side effects and requires extensive photosensitization preparation and thereby, patients have to be shielded from direct light exposure for 48 hours to prevent phototoxic reactions.^13^

A promising technique to visualize healthy and diseased parathyroid tissue has emerged in the use of intraoperative NIRF imaging using intravenously administered indocyanine green (ICG). This safe and rapidly evolving intraoperative imaging modality is described for use in a wide range of surgical procedures, such as identification of the biliary anatomy, assessment of anastomotic perfusion, and sentinel lymph node mapping.^14^, ^15^, ^16^ Suh et al. showed fluorescent PGs when using NIRF imaging with ICG in three dogs.^17^ Other studies showed the possibility of ICG fluorescent angiography to assess the remaining blood supply to the PGs in humans.^18^, ^19^, ^20^ Thus, NIRF imaging with ICG seems to be a promising technique to identify PGs during thyroid surgery and assess remaining perfusion of the PGs after thyroid removal.

The aim of this study was to assess the feasibility of NIRF imaging with ICG to identify PGs during thyroid surgery and to get an impression of the vascularization of the preserved PGs.

## 2 PATIENTS AND METHODS

This study was conducted at the Department of Surgery of the Maastricht University Medical Center (MUMC, Maastricht, Netherlands) between March 2017 and October 2017. The Medical Ethical Committee approved of this study, the study was conducted in full accordance with the ethical principles in the Declaration of Helsinki 2014^21^ and the protocol was registered on Clinicaltrials.gov with registration number NCT03012438.

**Figure 1:**
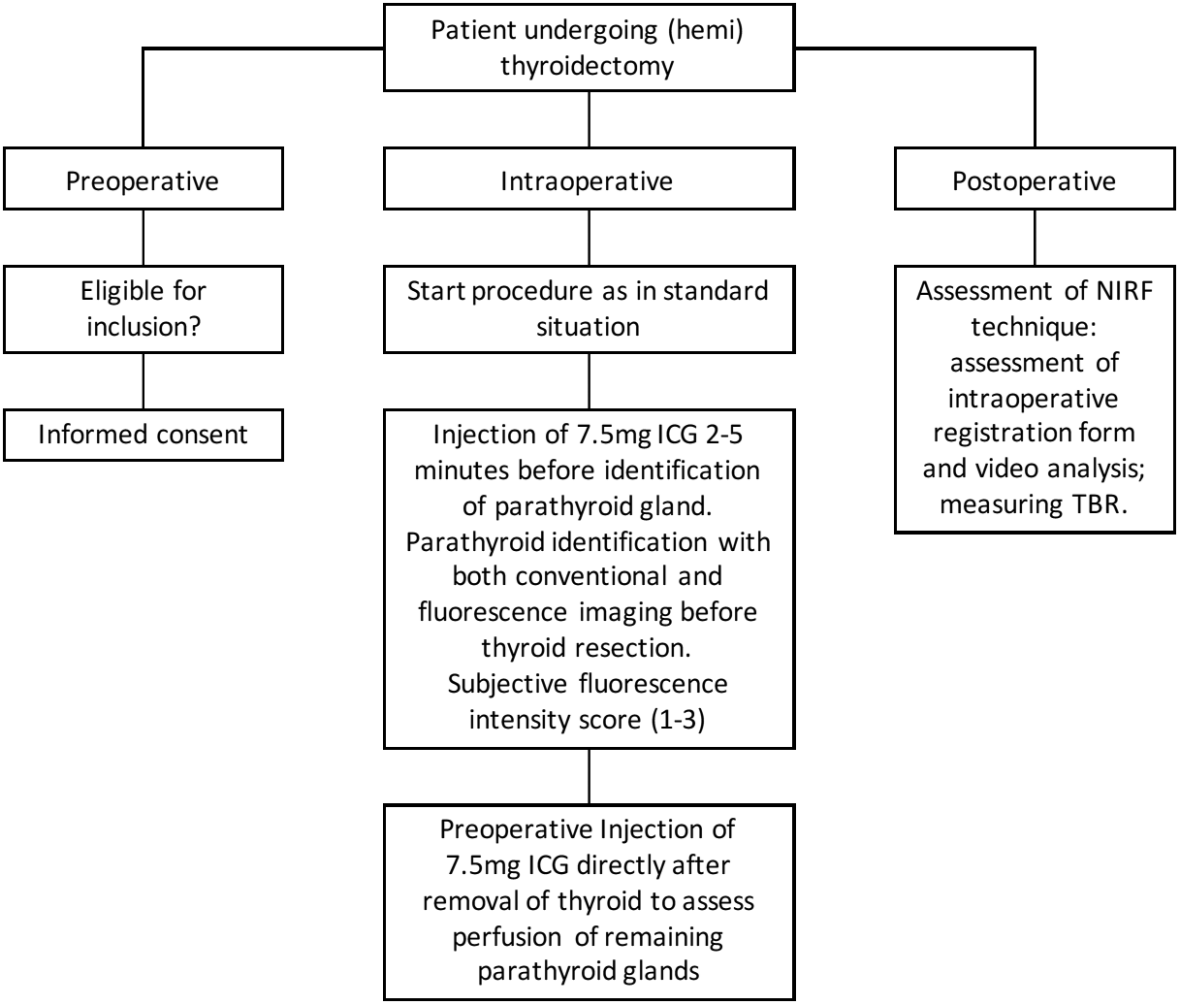
Flowchart of the study procedures

Consecutive men and women patients, aged 18 years and older, scheduled for elective total, hemithyroidectomy or completion thyroidectomy, with a normal liver and renal function and with written informed consent were eligible for inclusion. Subjects were excluded in case of known ICG, iodine, penicillin or sulfa‐hypersensitivity; pregnancy or breastfeeding; intravenous heparin injection in the last 24 hours (all contraindications for the use of intravenous ICG). A flowchart of the study procedures is shown in Figure 1.

### 2.1 Laparoscopic fluorescence imaging system and ICG administration

A commercially available laparoscopic fluorescence imaging system (Karl Storz GmbH & CO., Tuttlingen, Germany), including a plasma light guide, a 30° 10 mm laparoscope applicable for near infrared (NIR) light was used. Observations were done using white light, from the regular operation room‐lights and without camera, and in NIRF mode, using the dedicated system. Video recordings were made using the laparoscopic system.

ICG is a fluorescent contrast dye, which binds to plasma proteins and becomes fluorescent once excited by NIR light between about 820 and 840 nm. The dye was diluted with sterile water to a concentration of 2.5 mg/mL. Although having the laparoscopic imaging system in NIRF modus pointed at the thyroid, a bolus of 7.5 mg (3 mL) ICG (VERDYE, Diagnostic Green GmbH, Aschheim‐Dornacht, Germany) was administered intravenously at two different time points. The aim of the first bolus was to identify the PGs before removal of the (hemi‐)thyroid. The second bolus was used to assess vascularization of the PGs directly after thyroid resection. Both boluses of ICG were given just before using the NIRF‐modus of the fluorescence system.

In all total thyroidectomies, the surgeons searched for four PGs. In all hemithyroidectomies and completing thyroidectomies the surgeons searched for two PGs.

### 2.2 Data collection

The following intraoperative parameters were registered: whether PGs were identified, subjective fluorescence intensity of the PGs according to the surgeon, opinion of the surgeon about the usefulness of the technique, occurrence of intraoperative complications, the amount of time used for fluorescence imaging, and total surgical time.

The fluorescence intensity was scored by the performing surgeon on a 1‐3 grading scale: 1 means that the PG is black after injection of ICG, no fluorescence visible, 2 means that the PG is fluorescent, but no more than the surrounding tissue, and 3 if the PG is more fluorescent than the surrounding tissue.

Postoperatively, the thyroid specimen was checked by the pathologist for the presence of parathyroid tissue in the thyroid specimen. Perioperative complications were registered and fluorescence intensity on the video images was analyzed. The fluorescence intensity was analyzed using OsiriX Lite V8.5.2 Imaging software (Pixmeo, Geneva, Switzerland) to determine the target‐to‐background ratio (TBR). The TBR was defined as the mean fluorescence intensity (FI) of three points region of interest (ROIs) in the target (ie, PGs) minus the mean FI of three ROIs in the background (BG) (ie, surrounding tissue) divided by the mean FI of the BG ROIs. In formula TBR = (fluorescence intensity of target − fluorescence intensity of background)/fluorescence intensity of background.^22^ When this score is zero, there is no difference between the fluorescence intensity of the PG and the surrounding tissue. A TBR above zero indicates a more fluorescent PG compared to surroundings, and a TBR below zero indicates that the surroundings are more fluorescent compared to the PG.

### 2.3 Statistical analysis

Statistical analysis was performed using the Statistical Package for the Social Sciences v23 (IBM, Amonk, NY). A descriptive analysis was conducted for the patients' baseline characteristics and the following outcome measurements: total operation time, extra operation time, the presence of PGs in the resected thyroid, number of PGs identified in white light and NIRF, occurrence of perioperative complications, and the usefulness of the technique. The independent sample *t* test was used to assess whether there was a relation between the fluorescence intensity and factors, such as malignant or benign disease and normal or low calcium.

## 3 RESULTS

A total of 30 surgeries (hemi‐, total or completion thyroidectomy) in 26 patients were included. The majority of participants were female (73%) and the most common indication for thyroid surgery was suspected thyroid cancer (56.7%). Median body mass index (BMI) was 25.21 kg/m^2^. Further patient characteristics are shown in Table 1.

**Table 1:** Demographic and clinical details of included patients

	Patients (*n* = 30)^a^
Age (years)	56.3 ± 16
Weight (kg)	78.9 ± 17.6
BMI (kg/m^2^)	28.3 ± 6.9
Sex	
Male	8 (26.7)
Female	22 (73.3)
ASA‐classification	
1	3 (10)
2	25 (83.3)
3	2 (6.7)
Type of surgery	
Total thyroidectomy	6 (20)
Completion thyroidectomy	6 (20)
Hemithyroidectomy	18 (60)
Indication for surgery	
Multinodular goiter	5 (16.7)
Graves' disease	1 (3.3)
Suspected thyroid cancer	17 (56.7)
Proven thyroid cancer	7 (23.3)
Histology thyroid specimen	
Multinodular goiter	10 (33.3)
Thyroid cancer	11 (36.7)
Normal thyroid	3 (10)
Follicular adenoma	5 (16.7)
Thyroiditis	1 (3.3)

Data are presented as mean ± SD or number (%).

a The four patients with two included surgeries are counted twice.

### 3.1 Feasibility

No perioperative or postoperative complications due to the use of ICG occurred. Complications that occurred were an arterial bleeding after surgery for which reoperation was needed and a postoperative wound infection that required antibiotic treatment in another patient. The mean surgical time was 92 minutes (±32 minutes) for all surgeries. Herein, on average 5 minutes and 35 seconds (±94 seconds) were used for NIRF imaging. For a total thyroidectomy, mean surgical time was 126 minutes (±54 minutes); herein, 6 minutes and 27 seconds (±95 seconds) were spent using NIRF. A hemithyroidectomy took on average 83 minutes (±17 minutes) of which 5 minutes and 29 seconds (±55 seconds) were used for NIRF imaging. A completing thyroidectomy took on average 92 minutes (±22 minutes), 4 minutes and 51 seconds (±55 seconds) were spent using NIRF imaging.

### 3.2 Subjective usefulness of the technique

**Figure 2:**
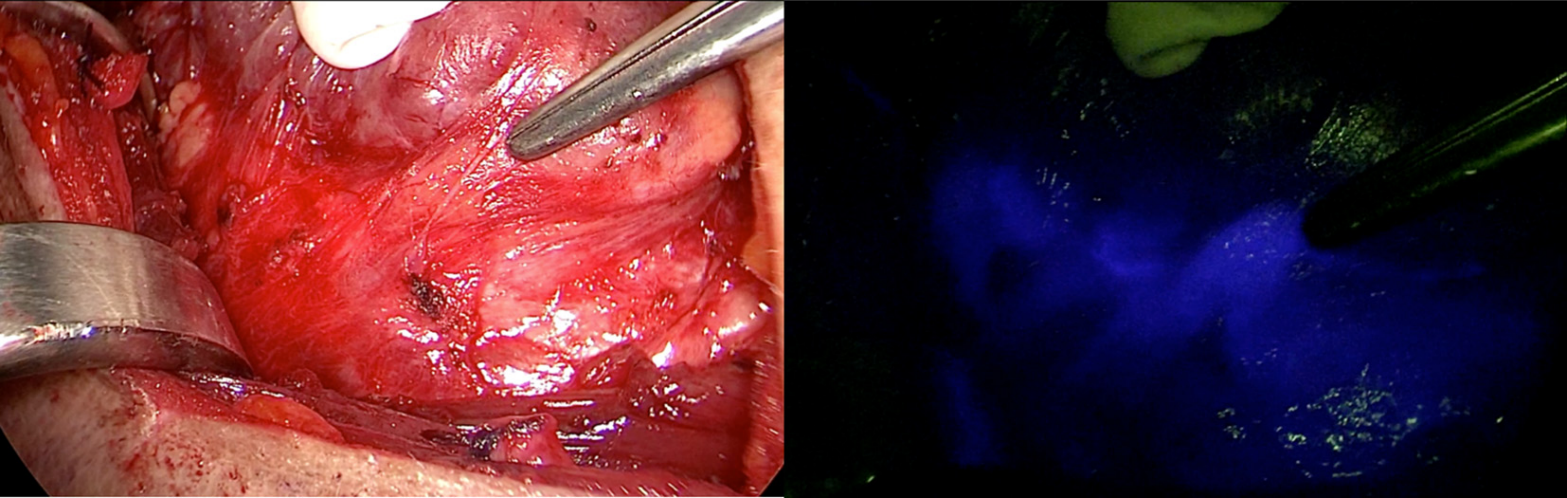
A (left), Suspected PG indicated by tweezers in white light. B (right), The suspected PG illuminated bright blue in fluorescence light. The NIRF signal outside the parathyroid gland is caused by the blood vessels toward and from the parathyroid gland [Color figure can be viewed at wileyonlinelibrary.com]

In 17 patients (57%), fluorescence imaging was rated as useful by the performing surgeon. In these patients, NIRF imaging provided certainty on both the localization and the preserved vascularization of the PGs. In two of the 17 patients, without the use of ICG, the surgical team would not have succeeded to identify a PG. In the remaining 15 patients, NIRF imaging was mainly helpful to confirm the suspicion of parathyroid tissue. An example of the obtained images is given in Figure 2 and an overview of reasons for usefulness or a lack of usefulness is given in Table 2.

**Table 2:** Usefulness of the technique

NIRF imaging considered useful	Total 17 patients
*Reasons*	*No. of patients*
PG was only identified using NIRF	2
Confirmation of suspected location PG	15
**NIRF imaging considered not useful**	**Total 13 patients**
*Reasons*	*No. of patients*
PGs already clearly visualized in white light, no extra certainty needed	6
PGs remained black after ICG administration	3
Surroundings too fluorescent to distinguish PGs	4

Three reasons were given for a lack of additional value in the remaining 13 patients. In five patients, the PGs were already clearly visualized in white light, no extra certainty was needed for these patients.

A second reason given for a lack of added value was that the PGs remained black after ICG administration in three patients. However, in these patients, the surgeon notes that it is possible that the PG was already dissected (*n* = 1 in which a PG was found in the histology assessment), or the thyroid was already manipulated extensively before using NIRF, possibly causing damage of the blood supply toward the PG (*n* = 2 hemithyroidectomies in which no calcium levels were determined due to the expected remaining two PGs in the other half thyroid). Therefore, although not for localization, the found black PG in these patients could have been of added value to indicate the surgeon that autotransplantation of this PG should be considered.

The third reason surgeons gave for a lack of added value was in two patients of lymphatic metastatic disease and two patients of nonmetastatic malignant cases: “the well‐vascularized cancerous tissue seemed to show misleading fluorescence intensity.” Because this tissue was so well vascularized, a lot of background fluorescence was present in these patients, making it difficult to distinguish between the cancerous tissue and the PGs based on NIRF imaging.

Further perioperative data are listed in Table 3.

**Table 3:** Overview included patients and fluorescence imaging

Surgery ID	Patient age (years)	Patient sex	Type of thyroidectomy	Extra surgical time (min:sec)	Before thyroid resection		After thyroid resection		Usefulness	Presence PG in specimen	Conclusion histology report thyroid
					Subjective FI	TBR	Subjective FI	TBR			
1	57	Female	Total	6:10	3	7.8	2	18.8	+	Yes	Multinodular goiter
2	43	Female	Hemi	4:45	3	30.5	3	12.8	+	No	Multinodular goiter
3	66	Female	Total	7:05	3	2.8	3	2.0	+	Yes	Papillary thyroid cancer
4	47	Female	Hemi	4:30	1	1.8	1	0.4	−	Yes	Follicular variant of papillary thyroid cancer
5	26	Male	Total	9:20	2	1.2	3	13.2	−	No	Papillary thyroid cancer
6	60	Female	Hemi	5:34	3	2.5	3	0.6	+	No	Multinodular goiter
7	58	Female	Hemi	8:00	3	2.1	2	0.5	+	Yes	Multinodular goiter
8	64	Male	Hemi	5:12	3	1.5	2	0.2	+	No	Follicular thyroid cancer
9	83	Female	Hemi	3:56	2		3	1.8	+	No	Multinodular goiter
10	70	Female	Hemi	8:40	3	12.1	1		−	No	Follicular hyperplasia
11	47	Female	Completion	5:45	2	0.9	1		+	No	Papillary thyroid cancer
12	75	Male	Total	5:50	2	0.5	2	0.4	−	No	Papillary thyroid cancer
13	70	Male	Total	4:56	2		3	1.5	+	No	Follicular hyperplasia
14	49	Female	Completion	−	3	1.7	3	1.7	−	Yes	Multinodular goiter
15	64	Male	Completion	5:43	2	0.7	3	0.6	+	No	Normal thyroid tissue
16	69	Female	Completion	4:55	2	0.5	2	1.6	−	No	Multinodular goiter
17	30	Female	Hemi	4:00	3	1.4	3	0.7	+	No	Follicular variant of papillary thyroid cancer
18	63	Male	Hemi	5:34	3	26.6	2		−	No	Follicular adenoma
19	43	Female	Hemi	5:00	3	0.8	3	1.8	+	No	Follicular adenoma
20	29	Female	Hemi	8:37	2	0.5	3	0.6	+	No	Papillary thyroid cancer
21	72	Female	Hemi	7:30	3	1.2	2	0.0	−	No	Multinodular goiter
22	29	Female	Completion	4:17	3	1.2	3	2.3	+	No	Normal thyroid tissue
23	76	Female	Hemi	4:15	3	−0.1	2	0.1	−	Yes	Papillary thyroid cancer
24	61	Male	Hemi	3:29	3	10.3	3	1.4	+	No	Follicular adenoma
25	54	Female	Hemi	3:57	3	3.2	3	1.6	+	No	Multinodular goiter
26	30	Female	Completion	3:38	3	1.6	2		−	No	Normal thyroid tissue
27	74	Female	Hemi	4:58	2	0.2	2	0.4	−	No	Multinodular goiter
28	54	Male	Hemi	6:09	3	0.9	3	0.8	+	No	Medullar thyroid cancer
29	66	Female	Hemi	4:40	1	−0.1	1	0.0	−	No	Lymphocytic thyroiditis
30	44	Female	Total	5:25	2		2	−0.3	−	Yes	Papillary thyroid cancer

FI, Fluorescence intensity; TBR, Target to background Ratio; PG, Parathyroid gland; + means considered useful, − means considered not useful.

### 3.3 Objective imaging and PG identification

In all total thyroidectomies and hemithyroidectomies, at least one PG was seen in either white or NIRF light before thyroid resection. In total, 41 PGs were visualized in white light in 25 patients, whereas with NIRF imaging in total 31 PGs could be identified in 23 patients. In three patients, no PG was identified during thyroid resection. All three patients underwent a completion thyroidectomy. In two hemithyroidectomy patients in whom initially no PG could be identified in white light, one PG was seen after the use of NIRF. In one patient, only one PG was seen in white light, whereas two were seen in NIRF light. These three patients underwent a hemithyroidectomy and no PGs were found in the resected material.

In nine patients, more PGs were visualized in white light. This group consisted of three total thyroidectomies and six hemithyroidectomies. In three of these patients, NIRF was used after extensive manipulation of the thyroid. In 18 patients, the same number of PGs was seen in white light as in NIRF light. In three patients, the number of PGs seen in white light is higher than expected, indicating a possible wrong identification of a PG. In NIRF imaging, a lower number of PGs were seen in two of these patients.

### 3.4 Histology

In seven patients, parathyroid tissue was found in the histopathology specimen. As can be seen in Table 4, in all these patients, only one PG was found in the specimen.

**Table 4:** Number of identified parathyroid glands in both white and NIRF light

Surgery ID	Type of thyroidectomy	Number of PGs to be expected	Number of PGs visible in white light	Number of PGs visible in NIRF	Number of PGs in resected thyroid
1	Total	4	4^a^	1	1
2	Hemi	2	2	1	0
3	Total	4	3	3	1
4	Hemi	2	2	0	1
5	Total	4	2	1	0
6	Hemi	2	2	2	0
7	Hemi	2	2^a^	1	1
8	Hemi	2	0	1	0
9	Hemi	2	2	2	0
10	Hemi	2	0	1	0
11	Completion	2	0	0	0
12	Total	4	1	1	0
13	Total	4	1	1	0
14	Completion	2	2^a^	2^a^	1
15	Completion	2	1	1	0
16	Completion	2	0	0	0
17	Hemi	2	2	2	0
18	Hemi	2	1	1	0
19	Hemi	2	1	2	0
20	Hemi	2	2	1	0
21	Hemi	2	2	2	0
22	Completion	4	1	1	0
23	Hemi	2	1	1	1
24	Hemi	2	1	1	0
25	Hemi	2	1	1	0
26	Completion	2	0	0	0
27	Hemi	2	1	0	0
28	Hemi	2	1	1	0
29	Hemi	2	1	0	0
30	Total	4	2	0	1
Total		72	41	31	7

a The tissue called parathyroid tissue in these patients could possibly be wrongly identified as PG tissue, because parathyroid tissue is found in the resected thyroid specimen.

Three out of these seven patients underwent a total thyroidectomy. In one of these patients, three PGs were seen both in white and NIRF light; in another patient, three PGs were seen in white light and one in NIRF; and in the third patient, two PGs were seen in white light and no PGs were seen with NIRF imaging. Another patient underwent a completion thyroidectomy in whom two PGs were seen in both white and NIRF light. The remaining three patients underwent a hemithyroidectomy. In all of them, two PGs were identified in white light; in one of those patients, no PG was seen with NIRF; and in the other two, only one PG was seen with NIRF imaging. Because in these patients parathyroid tissue was found in the resected thyroid, it is possible that what the surgeon called parathyroid tissue in white light actually was other tissue.

No significant correlation was found between the existence of parathyroid tissue in the histology specimen and the subjective fluorescence intensity during surgery.

### 3.5 Fluorescence intensity

Mean subjective fluorescence before and after thyroid resection was 2.53 (± 0.6) and 2.37 (± 0.7), respectively. Mean TBR before and after thyroid resection was 4.2 (± 7.6) and 2.5 (± 4.7), respectively. The subjective score on a scale 1‐3 was compared with the measured TBR. In two PGs, a subjective score of 1 (meaning PG is darker compared to surrounding) was given before thyroidectomy. In these patients, a mean TBR of 0.8 (±1.4) was found for those PGs, compared to a mean TBR of 0.7 (±0.3) in the PGs with a score of 2 (meaning PG as fluorescent as surrounding) and a mean TBR of 6.0 (±8.9) in the PGs in which a score of three was given (PG more fluorescent compared to surrounding). After thyroid resection in two patients, a score 1 was given, with a mean TBR of 0.2 (±0.2), a score of 2 resulted in a mean TBR of 2.4 (±6.1) and a subjective score of 3 in a TBR of 2.8 (±4.1). In none of the PGs which was scored as 3, a TBR below zero was measured. A higher TBR of the PGs before thyroid resection was seen in patients with benign pathology compared to patients with a malignancy (*P* = .044; CI = −9.6 to −0.14). This fluorescence intensity was on average 6.01 (±2.2) for benign histology and 1.1 (±0.8) in patients with malignant disease of the thyroid. The TBR after thyroid removal was with 3.0 (±5.1) and 1.8 (±4.0), respectively, not significantly different in patients with benign histology compared to malignant disease. No other factors contributing to a significant higher or lower TBR or subjective fluorescence intensity were found.

### 3.6 Function

In all patients who underwent total thyroidectomy, calcium supplementation was started postoperatively to prevent hypocalcaemia, according to standard care practice. Normal calcium levels were found in all patients 6 hours after surgery. In three patients who underwent total thyroidectomy, calcium ion levels dropped temporarily below the normal calcium‐ion value of our laboratory (normal range = 1.1‐1.3 mmol/L) the day after surgery. Calcium ion levels of these patients were 1.02, 1.03, and 1.09 mmol/L, respectively. Measured TBR in these patients after thyroid resection was lower than average with a mean TBR of 0.6 (±0.7), compared to a mean TBR of 5.1 (±2.4) in patients with a normal calcium ion level the day after surgery (*P* = 0.12). Calcium levels in these patients were restored after 2 weeks to 2.16, 2.30, and 2.32 mmol/L, respectively.

## 4 DISCUSSION

The main aim of this prospective, clinical study was to assess the feasibility of NIRF imaging with ICG to intraoperatively identify the PGs and to assess the perfusion after thyroid resection.

The use of NIRF imaging seems safe with no occurrence of complications due to the use of ICG in this study. The marginal extension of operation time in this study, related to the use of fluorescence imaging, will probably further decrease with growing experience. Eventually, it is expected that the total operation time will be shorter because of earlier identification of the PGs.

The NIRF technique has been evaluated frequently in previous studies for several purposes, but the experience with this technique in thyroid surgery is limited. In 2014, Suh et al.^17^ were the first to evaluate ICG for NIRF imaging of the PGs in an animal model. In three dogs, several doses of intravenous ICG ranging from 12.5 to 100 μg/kg were administered to illuminate the PGs. In all dogs, NIRF imaging was able to detect the PGs. The optimal dose of ICG based on these experiments was estimated to be 18.75 μg/kg. Sound et al.^23^ used NIRF imaging with ICG in three patients who underwent reoperative neck surgery for primary hyperthyroidism. In these series, initially, a dose of 5 mg ICG was given, and a second dose of 5 mg in one patient, and 3.25 mg in the other two patients. In all three patients, the PGs could be visualized after 2 minutes and stayed fluorescent up to 20 minutes. According to Yu et al.,^24^ a dose of 10 mg ICG might give better PG visualization intensity and time.

In the current study, PGs could be visualized in all 6 total thyroidectomies and all 18 hemithyroidectomies. In three of the six completion thyroidectomies, the PGs could not be detected in either NIRF or white light. This is unfortunate, as especially in these more difficult cases facilitation of PG identification is helpful. Possible explanations are the absence of PGs in the remaining thyroids or the perfusion of these PGs might have been cut off before performing fluorescence imaging. The absence of parathyroid tissue in the resected specimen shows that PGs in these patients were not located deeper in the thyroid.

Possible explanations for the inability to visualize PGs seen in white light with NIRF light are manipulation of the thyroid, causing damaged blood supply toward the PG. A second possible explanation is bright background signal due to highly vascularized malignant tissue. This is supported by the lower TBRs seen in patients with malignant histology conclusions. A third possibility is that those PGs seen in white light were not actually PGs, as parathyroid tissue was present in two patients in which more PGs were seen in white light compared to NIRF light. The anatomical location of the inferior PGs can make it hard to visualize the PGs. The PGs can be found around the crossing of the recurrent nerve and the inferior thyroid artery. As we use the NIRF angiography to identify the PGs, the nearby artery could confuse the location of the PG.

As the NIRF imaging is meant as a tool to assist the surgeon, we consider the scoring of the technique by the surgeon the most relevant. The operating surgeons were, therefore, asked to give a subjective score for the visibility of the PG compared to the surrounding tissue. This score ranged between 1 and 3. On average, a higher subjective score had a higher TBR although it had a broad SD. This means that the subjective observed score does not always correspond to the measured TBR. One possible explanation is the possible difference between the moving images during surgery based on which the subjective fluorescence intensity is determined and the screenshot in which the TBR is measured. Furthermore, the subjective score might be blurred by expectations, whereas the TBR is a more objective measurement, but influenced by the chosen regions of interest and background.

Also, the operating surgeons were asked whether they considered the used NIRF imaging of added value. In majority, NIRF imaging was considered of added value. In two patients, it is expected that no PGs would have been visualized without the use of ICG. In other patients, the added value consisted of reassuring the surgeon who is in doubt over the location of the PGs. Reasons for a lack of added value were mainly the surgeon who is already very certain about the location or other well‐vascularized tissue such as cancerous tissue that was also very fluorescent, causing the PGs not to be more fluorescent than the surroundings.

Apart from using ICG to identify the PGs, we also investigated the possibility to assess postoperative perfusion of the PGs. Earlier studies described the use of NIRF angiography of the PGs to estimate postoperative function.^18^ In our study, the post‐resection TBR was lower than before resection, suggesting confirmation of decreased vascularization. Lang et al.^18^ used NIRF for intraoperative angiography with ICG to evaluate postoperative functioning of the PGs. They included 70 patients and found that a high fluorescent light intensity was even more predictive for postoperative hypocalcemia than hypocalcemia immediately after operation. Vidal Fortuny et al.^25^ found that in all patients who had at least one well‐vascularized PG demonstrated by ICG‐angiography, the PTH levels 1 day postoperatively were normal. In our study, we did not measure postoperative PTH levels. This is only standard care in parathyroid surgery in our institute. However, we do have information on calcium levels. Postoperatively calcium supplementation was started 1 day after surgery in all patients who underwent total thyroidectomy as standard care. Blood is drawn the day after a total thyroidectomy, before calcium supplementation was started. A normal calcium‐ion level 1 day postoperatively would suggest normal parathyroid function, whereas calcium‐ion levels below normal can indicate reduced parathyroid function. In three patients, calcium levels were below normal value (1.1 mmol/L). In these patients, also TBR‐values seemed lower compared to patients with normal calcium levels the day after surgery, although not statistically significant. This is in line with the study performed by Zaidi et al.^20^ where a possible relation between fluorescence intensity and remaining function was found. In the current study, none of the patients developed permanent hypoparathyroidism.

In the current and the other available studies,^17^, ^20^, ^23^, ^25^ ICG is administrated just before aiming to visualize the PGs, as it is meant as angiography. After intravenous administration of the ICG, it takes about 30 seconds to reach the PG and thereby illuminate the gland of interest. We observed that not only the PG but also the thyroid gland and, to a lesser extent, the surrounding tissue are illuminated because of their vascularization, which causes ICG‐staining of the background and diminishes the contrast, both subjectively and objectively by the TBR. Previous studies have shown that ICG has the property of accumulation in pathological, hyperplastic PGs, which attributes to higher fluorescence intensity of the PG contrary to the surrounding tissues.^26^ It is not yet known whether this is also the case in normal PGs. Further studies are warranted with earlier administration of the ICG to evaluate its influence on contrast and accumulation in the normal PG.

The present study has some limitations. As the study was designed to be a feasibility study, a small number of patients were included, of which a minority of patients underwent a total or completion thyroidectomy. Therefore, only 12 patients were actually at risk of permanent hypoparathyroidism. The calcium‐ion level was measured postoperatively in the patients after a total thyroidectomy. However, a limitation of this study is that we could not histologically check the tissue we saw as parathyroid tissue in NIRF because of ethical reasons. We did, however, ask the pathologist to cut extra thin samples of the removed thyroid gland to check if parathyroid tissue was found in the extracted tissue.

In summary, this study showed that NIRF imaging with ICG for identification of the PGs during thyroid surgery is feasible. For the cases in which the ICG was helpful, it especially confirmed the suspicion of the presence of a PG as seen in white light, and helped to assess its vascularization after thyroid resection. In a minority of cases, NIRF identifies more PGs than white light imaging. However, it seems that in white light more wrongly identified PGs occurred. The findings of this pilot study confirm a possible role for ICG fluorescence imaging in the identification of the PGs, mainly in benign disease. Directions of further research should be aimed at improving the technique. Also, more in‐depth study of the correlation between the signal and the postop function of the PGs will further substantiate the relevance of the technique.

In conclusion, NIRF imaging with the use of ICG for intraoperative identification of the PGs and the assessment of its vascularization is feasible and safe and can provide more certainty about the location of the PGs.

## AUTHOR CONTRIBUTIONS

SE, TL, and NB performed the surgeries and edited the manuscript to its final version together with LS.

*Protocol for this study*: JvdB, LS, NB

*Patients inclusion and data analysis*: JvdB, LvK

*Coordinated the logistics of the study*: JvdB, LvK

*Wrote the first draft of the manuscript*: JvdB, LvK
